# Context-Dependent Effect of Glucocorticoids on the Proliferation, Differentiation, and Apoptosis of Regulatory T Cells: A Review of the Empirical Evidence and Clinical Applications

**DOI:** 10.3390/ijms20051142

**Published:** 2019-03-06

**Authors:** Luigi Cari, Francesca De Rosa, Giuseppe Nocentini, Carlo Riccardi

**Affiliations:** Section of Pharmacology, Department of Medicine, University of Perugia, Perugia I-06129, Italy; luigi.cari@hotmail.it (L.C.); francescaderosa94@libero.it (F.D.R.); carlo.riccardi@unipg.it (C.R.)

**Keywords:** glucocorticoids, regulatory T (Treg) cells, peripherally derived Treg (pTreg) cells, thymus-derived Treg cells (tTreg), Treg cell number modulation, human autoimmune diseases, human allergic diseases, desensitizing treatment, tolerogenic response

## Abstract

Glucocorticoids (GCs) are widely used to treat several diseases because of their powerful anti-inflammatory and immunomodulatory effects on immune cells and non-lymphoid tissues. The effects of GCs on T cells are the most relevant in this regard. In this review, we analyze how GCs modulate the survival, maturation, and differentiation of regulatory T (Treg) cell subsets into both murine models and humans. In this way, GCs change the Treg cell number with an impact on the mid-term and long-term efficacy of GC treatment. In vitro studies suggest that the GC-dependent expansion of Treg cells is relevant when they are activated. In agreement with this observation, the GC treatment of patients with established autoimmune, allergic, or (auto)inflammatory diseases causes an expansion of Treg cells. An exception to this appears to be the local GC treatment of psoriatic lesions. Moreover, the effects on Treg number in patients with multiple sclerosis are uncertain. The effects of GCs on Treg cell number in healthy/diseased subjects treated with or exposed to allergens/antigens appear to be context-dependent. Considering the relevance of this effect in the maturation of the immune system (tolerogenic response to antigens), the success of vaccination (including desensitization), and the tolerance to xenografts, the findings must be considered when planning GC treatment.

## 1. Introduction

Glucocorticoids (GCs) are widely used to treat both acute and chronic inflammatory conditions on account of their powerful anti-inflammatory and immunomodulatory effects on the activity and survival of immune cells and non-lymphoid tissues [1,2,3,4,5]. These effects of GCs vary across different types of tissues: GCs trigger apoptosis in some lymphocytes, but they provide protection against cell death in other lymphocytes or parenchymal cells in inflamed tissues. Array studies evaluating the mRNA levels in a diverse cell population have demonstrated that these differences have a genetic basis, as the majority of the genes modulated by GCs in a certain cell type are not modulated in the cells of other phenotypes [6,7,8,9,10,11,12,13]. When GCs are administered at clinical doses, their effects are the result of their interaction with their receptor (GR), which is expressed in nearly all cell types. The GR is located in the cytoplasm, where it is found in a multimeric chaperone complex or bound to other cellular structures, including kinases and transmembrane receptors [14,15,16]. The binding of GCs to GRs results in the activation of several pathways that promote their genomic and non-genomic effects [17,18,19,20,21,22,23].

GCs are also known for their immunosuppressive effects; as a result, they are administered after organ transplantation, during severe allergic reactions, or during autoimmune flare-ups. According to clinical observations, most of the therapeutic effects of GCs last for several days or weeks after their discontinuation [24,25,26], and one of the main long-term benefits of GCs is the transient shutdown of inflammation. The anti-inflammatory effects are brought about via modulation of the endothelial function and the inhibition of the production of several pro-inflammatory factors, including prostaglandins, nitric oxide, chemokines, cytokines, and their receptors [21,27,28]. These effects of GCs are evident in virtually all cells of the adaptive and innate immune system, and also exert immunosuppressive effects. 

In the last few years, several studies have indicated that the long-term effects of GC can also be attributed to their effects on the maturation and differentiation of several immune cells [29,30], such as dendritic cells (DCs) [31,32], macrophages [33,34,35,36] and thymocytes [37,38,39]. Of all the known cellular effects, the effects of GCs on T-cell survival, maturation, and differentiation are the most relevant. An important effect of GCs is evident in T-cell polarization. The high level of sensitivity of T-bet, which is selectively expressed in T helper (Th)1 cells, to GC inhibition favors Th2 development, particularly during long-term GC treatment [40,41]. Further, the GC-dependent upregulation of Itk, a Tec kinase that favors Th2 polarization, is potentially another polarization mechanism of GCs [42]. GCs also affect Th17 polarization by modulating cytokines such as interleukin (IL)-6, IL-17, and IL-23 [43,44]. Another GC-mediated effect on mature T cells is apoptotic cell death. The degree of activation and the timing of GC exposure (before, during, or after activation) render T cells sensitive or resistant to GC-induced apoptosis [45]. In addition, more recently, it has been demonstrated that GC treatment modulates the number of regulatory T (Treg) cells; thus, GC-mediated immune suppression may be partially achieved through an increase in Treg cell number or activity. 

In this review, we will present findings on the effect of GCs on the survival, maturation/differentiation, and growth of Treg cells in in vitro and in vivo models and in humans. In the next section, we will describe the different types of Treg cells and their functions. In the third section, we will present the in vitro findings reported so far on the effects of GCs on Treg cell expansion, and in the sections from the fourth to the seventh, the corresponding in vivo findings in healthy and diseased humans and animal models are described. The following sections review the findings reported on the effects of GCs under different contexts, such as during tolerogenic respiratory response to allergens, non-respiratory immune response to alloantigens or autoantigens, graft response, established autoimmune, alloimmune, and allergic diseases, and cancer. In the eighth section, the main pathways determining the different effects of GCs in conventional and Treg cells are reported. Finally, the concluding section will emphasize the most significant findings and their implications in the clinical context.

## 2. Treg Subsets and Functions

Treg cells are required for the control of immune homeostasis, which is defined as the dynamic balance between the activating and inhibitory effects of immune cells on the immune system. The disruption of homeostasis causes not only autoimmune diseases, but also inflammatory diseases, chronic infection, and other immune-related diseases (e.g., cardiovascular diseases and obesity) [46,47,48,49,50]. Cells of the innate immune system and endothelial cells participate in the activation of immune/inflammatory responses, and several subsets of cells belonging to the innate and adaptive immune systems participate in the negative regulation of immune response, including regulatory B cells, macrophages (specifically, M2 macrophages), myeloid-derived suppressor cells (MDSCs), tolerogenic DCs, non-αβ CD4^+^ T lymphocytes, CD4^−^CD8^−^ T cells, natural killer T cells, and CD8^+^ regulatory T cells. 

The most active subsets involved in the regulation of the immune response are αβ CD4^+^ Treg cell subsets. Treg cells exert regulatory effects via cell-to-cell contact, and also produce cytokines with immunosuppressive effects; in this way, they have a powerful effect on the microenvironment [51]. Indeed, according to several studies, the development and maintenance of autoimmune diseases, such as type 1 diabetes, rheumatoid arthritis, and relapsing-remitting multiple sclerosis, is associated with a decrease in the Treg population or a defect in the suppressive activity of Treg cells [52,53,54,55,56,57]. In other diseases, partial resistance of conventional T cells to Treg cells has been described [55,57,58,59]. In both instances, an increase in the number of fully active Treg cells may prove beneficial. However, in the case of cancer, an increase in the number of Treg cells in the tumoral microenvironment favors the development of tumoral cells and protects them from immune attack [60,61,62,63,64,65]. These findings indicate that while Treg cell expansion is beneficial in the context of certain diseases, it is detrimental and favors disease pathogenesis in the context of cancer. In addition to these findings, in the last few years, Treg cells have been shown to play a crucial role in tissue repair and maintenance, too [66,67,68]. This mechanism is distinct from their suppressive role, and is elicited by IL-18, IL-33, and amphiregulin. 

Treg cells are classified as thymus-derived Treg (tTreg) cells and peripherally-derived Treg (pTreg) cells. tTreg cells were originally identified as CD4^+^CD25^+^ T cells in healthy mice; it was only a few years later that tTreg cells were also found to express the transcription factor forkhead box P3 (FoxP3) [69,70,71]. The exclusive use of CD25 as a marker of tTreg cells might be misleading, as CD25 is also expressed by CD4^+^ conventional T cells following activation. Some authors have identified tTreg cells as CD25^high^ (but activated CD4^+^ T cells may be CD25^high^) or, better, as CD4^+^CD25^+^CD127^low/−^ or CD4^+^CD25^+^FoxP3^+^ [46]. In addition, tTreg cells are characterized by many other surface markers, including GITR, CTLA-4, HLA-DR, and CD39 [55,72,73]. It is yet to be established whether differences in the expression of these markers can be used to differentiate between different tTreg subsets or tTreg cells, which exhibit specific activity at specific time points. This is because the findings reported so far are contradictory in this regard. For example, some reports have suggested that FoxP3 is not necessarily associated with regulatory functions and, at least in some instances, it acts as an activation marker [74,75,76]. Such contradictory results have also been reported regarding the suppressive capacity of human-activated CD25^+^FoxP3^+^ cells [77,78,79,80,81,82,83,84]. tTreg cells that exit the thymus are naïve (CD45RA^+^), but when they come into contact with antigens in the periphery, they become memory T cells (CD45RA^−^, sometimes identified as CD45R0^+^). 

By definition, pTreg cells are memory T cells (CD45RA^−^ or CD45R0^+^), as they are derived from activated CD4^+^ conventional T cells in the presence of appropriate signals, such as transforming growth factor (TGF)-β and interleukin (IL)-10, in the microenvironment [85,86,87,88]. Unlike tTreg cells, pTreg cells may not express CD25 and FoxP3, and do not have a homogeneous phenotype or identical functions. The best characterized pTreg subsets are T helper (Th)3 cells (CD25^+^FoxP3^+^), T regulatory type 1 (Tr1) cells (CD25^−^FoxP3^−^), and CD25^−/low^FoxP3^−/low^GITR^+^ (GITR single-positive or GITRsp) cells. Th3 cells develop after exposure to oral antigens, and have been shown to inhibit the development of experimental autoimmune encephalomyelitis [89,90]. Tr1 cells are induced under in vitro settings, and they produce IL-10 and inhibit inflammatory responses in the colon and central nervous system [91,92,93]. GITRsp cells are expanded in the tumor microenvironment and represent a homeostatic response in autoimmune diseases [58,94,95,96]. In addition to these, there are other pTreg cell subsets, such as those that develop after epicutaneous immunization with autoantigenic peptides and inhibit experimental allergic encephalomyelitis, those that develop after respiratory exposure to antigens and inhibit the development of allergen-induced airway hyper-reactivity, and those that develop during Th1-polarized immune responses to ovalbumin (OVA) and express ICOS, IL-10, and interferon-γ [97]. Besides GITR and ICOS, many other surface markers may be used to characterize pTreg subsets, including LAG-3, CTLA-4, and CD39 [51,55].

## 3. Effect of GCs on Treg Cell Number: In Vitro Findings

### 3.1. Effects on non-Activated Treg Cells

Several in vitro studies have evaluated the effect of GCs on tTreg cells and the differentiation of conventional T cells into pTreg cells. From the studies that have investigated the effects of the GC dexamethasone on total CD4^+^ T cells or even peripheral blood mononuclear cells (PBMCs), it is unclear whether dexamethasone affects the differentiation of CD4^+^ cells into pTreg cells or the expansion of tTreg and pTreg cells present at the original site. Moreover, the results are contradictory [98,99,100,101]. Pandolfi et al. [100] used the 10^−7^ M dexamethasone concentration, which is equivalent to the in vivo concentration of GC after the administration of a therapeutic dose of GC or following a highly stressful event [38]. Thus, their findings suggesting that unstimulated Treg cells undergo apoptosis following GC treatment in a dose-dependent and time-dependent manner may represent what happens in vivo. However, in some experimental settings [98,99], Treg cells appear to be less sensitive than non-Treg T cells to GC-induced apoptosis, resulting in a relative increase of Treg cells as compared to non-Treg cells.

### 3.2. Effects on Activated Treg Cells

It is reasonable to hypothesize that in a patient affected by an inflammatory/autoimmune disease, the majority of T and Treg cells are activated. Therefore, it is interesting to understand the effect of GC treatment on activated T and Treg cells. 

Some studies deal with the short-term effect of GC following T cells’ activation in human and murine cells. However, reported results are conflicting [101,102,103]. This is because the cellular response to the activation stimulus and the accumulation of IL-2 require times, also depending on the experimental settings [100]. Therefore, the effect of GCs on (still) non-activated cells (despite formally activated) may be more relevant than the effect of GCs on activated cells. Therefore, in order to understand the effects of GCs on activated Treg cells, it is much better to consider the long-term effects of GCs on activated T and Treg cells.

Several studies have investigated these effects. In one of these studies, Karagiannidis et al. examined the effect of dexamethasone (10^−7^ M) on the differentiation of human naïve CD4^+^ T cells treated with anti-CD3/anti-CD28/anti-CD2 antibodies plus IL-2 [98]. After eight days, the CD4^+^ T cells showed a seven-fold increase in FoxP3 mRNA expression; this indicates that GCs favor the differentiation of conventional T cells into pTreg cells. Another study on humans confirmed these findings, demonstrating that high concentrations of the GC fluticasone propionate skew the cytokine profile of allergen-driven CD4^+^ T cells from atopic subjects toward a T regulatory phenotype, with the elevated production of IL-10 (i.e., Tr1-like) [104]. Prado et al.’s study further confirms the Karagiannidis et al. study working with human purified T cells [102]. On day 14, flow cytometry analysis revealed that there was an increase in FoxP3 expression (about two-fold) in the dexamethasone-treated cells as compared to the untreated cells. Moreover, dexamethasone treatment resulted in an increase in the number of CTLA-4 cells and a decrease in the number of CD69^+^ cells in the CD25^+^ cell population. Based on these findings, the authors concluded that GCs resulted in the expansion of Treg cells defined as CD4^low^CD25^high^CD69^low^GITR^+^CD45RO^+^CD127^−/low^CTLA-4^+^FoxP3^+^. The same study investigated the effects of dexamethasone in the TGF-β-induced polarization of CD4^+^CD25^−^ T cells (but not naïve CD4^+^ T cells) [102]. As expected, TGF-β induced an increase in the level of FoxP3 (both mRNA and protein), but dexamethasone induced a further increase in the FoxP3 level, which was even higher than that in the purified tTreg cells (CD25^high^). In another study, Chen et al. demonstrated that dexamethasone plus IL-2 treatment resulted in an increase in GITR expression in CD4^+^CD25^+^ T cells [101]. Interestingly, GITR had been originally cloned because it was overexpressed by a hybridoma cell line treated with dexamethasone, and GITR is considered to be a marker of activated Treg cells [105,106]. 

However, the expansion of Treg is dependent on the type and strength of activation. In fact, in another long-term study, when human T cells, co-cultured with the other PBMC, were activated with the tetanus antigen plus IL-2, dexamethasone (10^−7^ M) decreased the levels of FoxP3 mRNA following long-term culture (11 days) [103].

Unexpectedly, the increased expression of FoxP3 in the population observed in human T cells did not correlate with the increase in the suppressive activity of the cell population [102]. A similar observation was reported by Chung et al., who demonstrated that in CD4^+^CD25^−^ T cells treated with IL-7, dexamethasone resulted in an increase in the number of cells expressing CD25, but not in the suppressive activity of the population [107].

However, the lower level of suppressive activity of expanded/differentiated Treg cells induced by GCs may be an in vitro artifact, and is contradicted by a study on murine T cells demonstrating that dexamethasone-treated CD4^+^CD25^+^ T cells retain suppressive effects that are equivalent to those of freshly isolated CD4^+^CD25^+^ Treg cells [101]. In our opinion, the best way to test the suppressive activity of GC-expanded Treg cells is to perform in vivo studies.

In conclusion, GCs favor the differentiation of activated CD4^+^ naïve and conventional T cells into Treg cells. Moreover, Treg cells activated by a strong stimulus do not undergo GC-induced apoptosis. For these reasons, Treg cells increase in number relative to conventional T cells. Their suppressive activity after the expansion is still a matter of debate.

### 3.3. Effects on non-T Immune Cells

The promotion of Treg expansion/differentiation by GCs is also dependent on the effects of GCs on other cells of the immune system. At the beginning of this century, it was demonstrated that GC-treated human DCs produce IL-10, and DC-derived IL-10 induces pTreg differentiation [108,109]. Barrat et al. stimulated murine and human naïve CD4^+^ T cells with vitamin D3 and dexamethasone (5 × 10^−8^ M) in the presence of antigen-presenting cells and OVA, and demonstrated that the treatment promotes the differentiation of CD4^+^ T cells into IL-10-producing Tr1 cells [91]. Further, Xystrakis et al. demonstrated that one week of the culture of CD4^+^ T cells with anti-CD3 antibodies, antigen-presenting cells, IL-2, and IL-4 in the presence of dexamethasone and vitamin D3 promoted an IL-10-expressing phenotype [110]. In the following years, several other studies confirmed the role of GCs (independently or in association with other factors) in promoting the differentiation of tolerogenic DCs and the role of DC-derived IL-10 in the differentiation of conventional T cells into pTreg cells, including Tr1 cells [32,111,112].

Stary et al. demonstrated that human GC-treated Langerhans cells exhibit a more immature phenotype and higher intracellular amounts of TGF-β, both of which are conditions that favor the expansion of Treg cells [113]. Indeed, an enhancement of functionally suppressive FoxP3^+^ T cells was observed when T lymphocytes were incubated with GC-treated Langerhans cells. Other studies have demonstrated that MDSCs play a role in the potentiation and increase in the number of Treg cells. In fact, dexamethasone promotes the suppressive function of human-derived MDSCs in vitro, and favors the expression of IL-10 and TGF-β [114,115]. Moreover, in mice, heart graft survival promoted by dexamethasone correlated with an increase in Gr-1^high^CD11b^+^ MDSCs and CD3^+^CD4^+^FoxP3^+^ Treg cells, but the administration of anti-Gr-1 antibody in dexamethasone-treated mice shortened heart graft survival and reduced the number of Treg cells [116].

**Figure 1:**
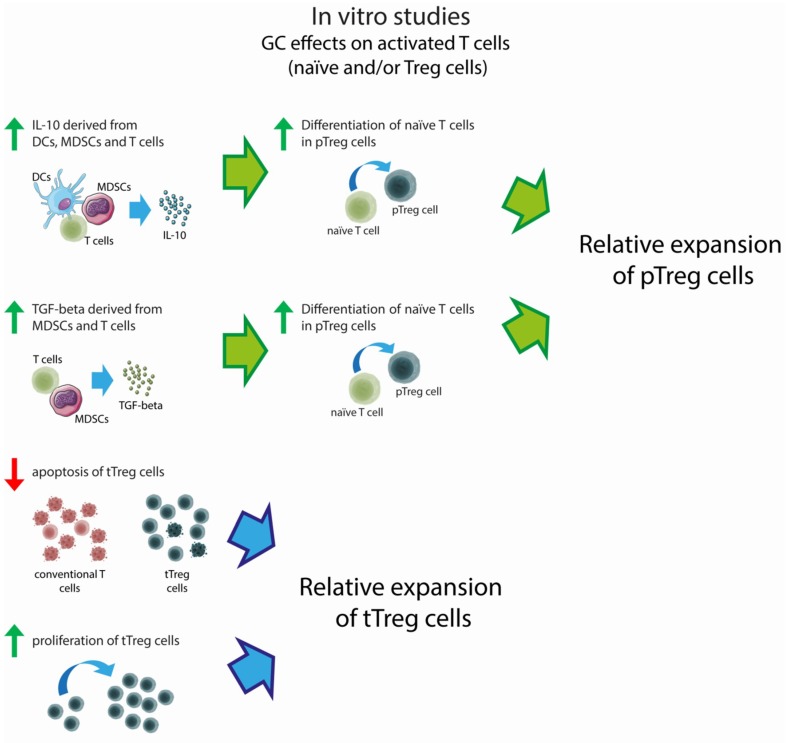
Effects of in vitro glucocorticoid (GC) treatment on activated T cells. Red arrows indicate lower levels of apoptosis as compared to conventional T cells; green arrows indicate increased cytokine production, proliferation, and differentiation.

In conclusion, there is overwhelming evidence that GCs favor Treg expansion in activated T cells, mainly because they promote the differentiation of CD4^+^ T cells into pTreg cells. This effect is also due to the GC-dependent maturation/expansion of DCs, Langerhans cells, and MDSCs, promoting the differentiation and expansion of pTreg cells indirectly. The possible negative effects of GCs in resting Treg cells may mean that GCs determine a decrease of Treg cells when given to healthy subjects. The in vitro effects of GC are summarized in Figure 1.

## 4. Effect of GCs on Treg Cell Number: In Vivo Findings in Healthy Humans and Animals

More than 10 years ago, it was demonstrated that a single dose of dexamethasone induced an increase in the proportion of murine CD4^+^CD25^+^ Treg cells in thymocytes and splenocytes. The increase was not due to an increase in the number of Treg cells, but rather to a decrease in the number of non-Treg T cells [101]. The percentage increase in the number of CD4^+^CD25^+^ T cells in the thymus was more impressive (six-fold increase) when multiple doses of dexamethasone were administered. However, in other lymphoid organs, three consecutive doses of dexamethasone resulted in a much lower (1.3 to 1.4-fold) increase in the percentage of Treg cells. Other data on mice confirmed these findings, even when Treg cells were considered to be CD4^+^CD25^high^ and express FoxP3, IL-10, and TGF-β [117,118]. Interestingly, Ugor et al.’s study indicated that murine tTreg cells are resistant to dexamethasone-induced apoptosis, while pTreg cells are not, possibly suggesting that the increased proportion of Treg following GC treatment is due to the resistance of tTreg to GC-induced apoptosis [118].

Sbiera et al. evaluated the absolute number and percentage of CD4^+^ T cells in the Treg cell population following the long-term dexamethasone treatment of mice and humans [119]. In mice, the absolute number and percentage of Treg cells in the blood had decreased. In contrast, in humans, long-term dexamethasone treatment resulted in a significant increase in the number of CD4^+^ T cells and Treg cells, including CD4^+^CD25^high^FoxP3^+^, CD4^+^CD25^high^CD127^−^, and CD4^+^CD25^high^CTLA-4^+^ Treg cells. This means that human peripheral CD4^+^ and Treg cells do not undergo apoptosis following GC treatment, unlike the findings in mice. However, in the CD4^+^ cell population, the proportion of CD4^+^CD25^high^FoxP3^+^ and CD4^+^CD25^high^GITR^+^ cells had slightly decreased, the proportion of CD4^+^CD25^high^CD127^−^ cells had slightly increased, and the proportion of CD4^+^CD25^high^CTLA-4^+^ cells remained unchanged. This means that in healthy humans, GC treatment does not substantially change the ratio of CD4^+^ conventional T cells to Treg cells. However, the CD4^+^CD25^high^GITR^+^ Treg cell population was investigated using an anti-GITR antibody that has very low sensitivity (Cari and Nocentini, unpublished data), so data concerning this subpopulation should not be considered.

Since in vitro studies have demonstrated that murine Treg cells are more resistant to dexamethasone-induced cell death and are protected by IL-2 [101], Chen et al. tested the in vivo effects of the co-administration of dexamethasone and IL-2 on Treg cells [120]. The percentage of CD4^+^CD25^+^ T cells in the spleen, inguinal, and mesenteric lymph nodes increased by 88%, 25%, and 33%, respectively (*p* < 0.01), after a single IL-2/dexamethasone dose, and by 180%, 75%, and 95% after five days of daily treatment. The CD4^+^CD25^+^ to CD4^+^CD25^−^ cell ratio also increased. The increase was not only due to the diminished number of CD4^+^CD25^−^ T cells, but also due to the enhanced number of CD4^+^CD25^+^ T cells (e.g., 200% in the spleen). The authors demonstrated that the increase in the percentage of CD4^+^CD25^+^ T cells was due to the expansion of tTreg cells and not due to the differentiation of conventional T cells into pTreg cells, and that expanded Treg cells expressed FoxP3 and exhibited a regulatory phenotype.

Thus, similar to the in vitro studies, the in vivo studies on the effect of dexamethasone administered alone and in combination with IL-2 also demonstrate that the GC-induced expansion of Treg cells is more relevant when Treg cells are activated. The activation of Treg cells induced by IL-2 in the experimental setting might be similar to the activation of Treg cells observed in an inflammatory microenvironment. In fact, this has been confirmed in an interesting study performed on horses [121], where the authors collected bronchoalveolar lavage fluid (BALF) from asthmatic and non-asthmatic horses before and after treatment with dexamethasone. At baseline, the percentage of FoxP3^+^ cells in CD4^+^ cells in the BALF was higher (although not significantly) in asthmatic horses than non-asthmatic horses. After two weeks of daily treatment, the percentage of FoxP3^+^ cells was decreased (although not significantly) in the non-asthmatic horses, and was increased significantly in the asthmatic horses as compared to the respective baseline data. Another study demonstrated that in patients affected by autoimmune diseases of the connective tissue, the number of Treg cells was lower when the patients were treated with both GCs and immunosuppressive drugs [122]. This data together with those presented in Section 6 confirms that the effect of GCs on Treg cells when they are not activated is the opposite of the effects of GCs on activated Treg cells. 

In conclusion, the findings discussed here indicate that the induction of Treg cell expansion by GCs in healthy humans and animals depends on the activating co-treatment conditions and whether or not the Treg cells are activated during the disease. In particular, Treg cells expansion is observed when T cells are activated by a strong stimulus. However, exceptions to this general rule are observed, as reported in the following paragraphs. The main data reported by the in vivo studies on the effects of GCs on Treg number are reported in Table 1; Table 2.

## 5. Effect of GCs on Treg Cell Number: In Vivo Findings during Development of Immune Response

### 5.1. Effects during the Tolerogenic Respiratory Response to Allergens and Sensitization to Respiratory Allergens

It is well known that during maturation of the immune system in infancy and in response to allergens used in the allergen immunotherapy, the increase of Treg cells plays a pivotal role. For example, the treatment of mice with OVA and heat-killed *Listeria monocytogenes* as an adjuvant induces the differentiation of conventional CD4^+^ T cells into pTreg cells that express FoxP3 and ICOS and produce both IL-10 and interferon-γ. This pTreg subset inhibits the development of allergen-induced airway hyperreactivity [97]. Stock et al. demonstrated in mice that treatment with GCs counteracts the protective effects of respiratory tolerance on the development of airway hyperreactivity by inhibiting the development of the above-mentioned pTreg cells [143]. This effect is attributable to the GC-induced inhibition of IL-10 production by DCs. Therefore, GCs, while downregulating Th2-driven allergic pulmonary inflammation, may inhibit respiratory tolerance, which is thought to limit immune responses against the large quantities of innocuous antigens suspended in inspired air that enters the lungs [144,145].

A study evaluated the effect of systemic and topical GCs in the sensitization of mice to house dust mite [125]. Dexamethasone administered at a dose of 1 mg/kg per day inhibited house dust mite-evoked lung inflammation and airway hyperreactivity, but both dexamethasone and budesonide (7.5 mg/mL) induced a reduction in Treg cell (CD4^+^CD25^+^FoxP3^+^) numbers in the lungs and lymphoid organs of allergen-challenged mice. In particular, dexamethasone administration during the entire sensitization procedure (three weeks) inhibited the airway infiltration of Treg cells in allergen-challenged mice at the peak of lung inflammation and during the resolution, as evaluated in BALF and lung tissue. Interestingly, the percentage of IL-10-producing CD4^+^ cells in BALF was decreased in the animals treated with systemic dexamethasone or topic budesonide; this indicates that the decrease in the number of Treg cells is due to the dexamethasone-dependent inhibition of IL-10 expression. 

Other studies have evaluated the effect of systemic and topical GCs in the OVA-induced model of asthma and a rhinitis model, with similar results [123,126,127,146]. For example, the study by Zuska-Prot et al. [123] demonstrated that during the first days of the challenge, a concomitant reduction in the absolute number of CD4^+^CD25^+^FoxP3^+^ Treg cells and CD4^+^ conventional T cells was observed in the lungs and mediastinal lymph nodes of GC-treated mice, but the decrease in Treg cells was more pronounced, possibly resulting in a decrease in the percentage of Treg cells. The data are summarized in Table 1. Therefore, despite one contrasting result being published [127], the reported data suggested that in most experimental conditions, GC decreased the Treg cell number during the tolerogenic respiratory response to allergens and sensitization to respiratory allergens, suggesting that a similar effect might happen in humans.

Indeed, an interesting study confirmed that the findings can be extrapolated to humans [147]. The authors treated asthmatic children who were allergic to house dust mites with the desensitization treatment, consisting of the injection of increasing doses of the specific allergen, and demonstrated that FoxP3 mRNA in PBMC and the percentage of FoxP3^+^ cells in CD4^+^CD25^+^ cells increased significantly after one year from the start of treatment (build-up and maintenance phases). On the contrary, no increase in FoxP3 mRNA expression was observed in asthmatic children treated with oral prednisone or oral prednisone plus vitamin D3 before subcutaneous injection of the desensitizing agent during the build-up phase. The increase in the percentage of FoxP3^+^ cells in the CD4^+^CD25^+^ cell population was much lower (1.7-fold increase) in the prednisone-treated and immunotherapy-treated groups than in the GC-untreated immunotherapy-treated group (3.7-fold increase). Thus, the findings of this study suggest that the administration of GCs during allergen immunotherapy is not recommended, because it does not allow the expansion of Treg cells, which may be crucial for the long-term effect of the treatment.

### 5.2. Effect during Immune Response against Alloantigens or Autoantigens in Systems Other Than the Respiratory System

Vaccination for protection from autoimmune and alloimmune diseases (desensitization) is a popular concept in the field of immunotherapy. More than 10 years ago, Kang et al. sensitized mice to OVA by the subcutaneous injection of OVA and evaluated the OVA-induced delayed-type hypersensitivity to OVA by rechallenge at a footpad [148]. Then, mice were treated with an OVA-derived, MHC II-restricted peptide (OVA_323–339_). When mice were treated with OVA_323–339_ or dexamethasone alone, delayed-type hypersensitivity was still present. On the contrary, when OVA_323–339_ was administered in association with dexamethasone, swelling of the footpad was reduced. In these animals, the Treg cell (CD4^+^CD25^+^FoxP3^+^) percentage in the blood and draining lymph node (popliteal) was almost doubled. These findings demonstrate that the GC–peptide association can exert a tolerogenic response, which is partly caused by the increase in Treg cell number.

The same group of authors conducted desensitization experiments in the diabetes-prone NOD mice, which are considered a model of autoimmune diabetes, by treating them with dexamethasone and the insulin-derived, MHC II-restricted peptide antigen B:9–23 [148]. Mice treated with either dexamethasone or B:9–23 showed a delay in the development of diabetes, but mice treated with both dexamethasone and B:9–23 remained disease-free during the entire period of the experiment. In the mice that survived, Ag-specific Treg cells with greater sensitivity to restimulation with B:9–23 were detected in the spleen. The effects of dexamethasone in association with the insulin antigen were confirmed in a similar study, showing an increase of CD4^+^CD25^+^FoxP3^+^IL-10^+^ and CD4^+^CD25^+^FoxP3^+^ Treg subset cells in mice that were co-treated with dexamethasone and the insulin B:9–23 peptide [130]. 

Recently, Chen et al. demonstrated that dexamethasone potentiates the immune response and favors the expansion of CD4^+^FoxP3^+^ Treg cells after HSP60-targeted immunization in the ApoE^−/−^ mouse model of atherosclerosis [128]. Further, even in a murine model of colitis (induction by dinitrobenzene sulfonic acid—DNBS), dexamethasone in association with TGF-β increased the number of Treg cells slightly [129]. Thus, even though GCs can cause a decrease in the Treg cell number in some circumstances (such as during the development of allergic response/desensitization to a respiratory allergen, as described in Section 5.1), in the presence of different antigens and/or in other tissues, GCs seem to induce an increase in the Treg cell number and favor the tolerogenic response.

### 5.3. Effect during Graft Response

GCs are used to prevent and treat organ transplant rejection. As is the case with the other effects, these effects are dependent on the effects of GCs on several cells of the immune system, as well as the production of immunosuppressive cytokines and the inhibition of pro-inflammatory cytokines. 

Some studies have evaluated the effects of GCs on Treg number in the context of graft rejection. In one such study, Luan et al. found that the in vivo levels of MDSCs and Treg cells were increased in kidney transplantation patients who received steroid-based immunosuppressive therapy, and that MDSCs mediated in vitro Treg cell expansion [149]. Further, Nakao et al. recently demonstrated that dexamethasone prolongs cardiac allograft survival in a murine model [116]. As mentioned before, these effects were due to an MDSC-dependent increase in Treg cells. In fact, the killing of MDSCs shortened mouse heart graft survival and reduced the number of Treg cells in dexamethasone-treated mice. These findings indicate that an increase in the number of Treg cells is crucial for the GC-dependent protection of allografts.

Seissler et al. evaluated how methylprednisolone bolus therapy altered the percentage of Treg (CD4^+^FoxP3^+^CD127^low/−^) cell subsets in transplant patients with biopsy-proven rejection [133]. They demonstrated that GC therapy resulted in an increase in the percentage of HLA-DR^+^CD45RA^−^ Treg cells. Interestingly, the greatest increase was detected in the HLA-DR^high^CD45RA^−^ Treg cells that showed the highest level of suppression. Further, these effects were temporarily and closely associated with the duration of the bolus therapy.

Thus, the few studies reported so far suggest that the GC-dependent prevention of allograft rejection is attributable, at least in part, to the expansion and activation of Treg cells.

## 6. Effect of GCs on Treg Cell Number: In Vivo Findings in Established Autoimmune, Allergic, and (auto)Inflammatory Diseases

Several studies on both murine models and human diseases demonstrate that one of the beneficial effects of GCs is the induction of Treg cell expansion in autoimmune, allergic, and autoinflammatory diseases, as shown in Table 1 (animal models) and Table 2 (patients).

### 6.1. Effects in Autoimmune Diseases

In 2006, Suarez et al. reported that systemic lupus erythematosus (SLE) patients had a higher percentage of CD4^+^CD25^high^ Treg cells than healthy subjects; however, a sub-analysis of the data demonstrated that the higher percentage of Treg cells (about two-fold) was present only in the patients treated with GCs [136]. Indeed, 70% of the patients with a Treg cell increase >54% (as compared to the control patients) were treated with GCs. Azab et al. confirmed these findings, and demonstrated a strong correlation between Treg cell percentage and GC dose [134]. Further, in new-onset SLE patients with active disease, treatment with GCs and cyclophosphamide resulted in a significant increase in the percentage of CD4^+^CD25^high^ cells (about 1.3-fold), but not in the percentage of CD4^+^FoxP3^+^ cells [137]. In Mathian et al.’s study, PBMCs that were positive for FoxP3 were divided into three subsets: FoxP3^low^CD45RA^+^ (naïve Treg cells), FoxP3^high^CD45RA^−^ (effector Treg cells), and FoxP3^low^CD45RA^−^ cells (non-regulatory FoxP3^low^ T cells). They demonstrated that in new-onset SLE patients with active disease, treatment with GCs increased the number of effector Treg cells by 4.6-fold at three days following intravenous high-dose methylprednisolone treatment, but the percentage of these cells returned to baseline eight days after high-dose methylprednisolone treatment. On the contrary, the percentage of naïve Treg cells was not altered after treatment, and the percentage of non-regulatory Foxp3^low^ T cells decreased only slightly [135].

In patients with immune thrombocytopenic purpura (ITP), GCs induced an increase in the FoxP3^+^ Treg subset (more than two-fold) following four days of high-dose dexamethasone administration, and in the CD25^+^CD127^−^ Treg subset (less than two-fold), at day 14 [138,139]. However, the CD25^+^CD127^−^ Treg subset decreased to the baseline levels at day 28 [138]. Similar data were obtained more recently by other authors [99].

In conclusion, it seems that in SLE and ITP, GCs exert their therapeutic effects by inducing an increase in the percentage of Treg subsets. 

### 6.2. Effects in Asthma

With only one exception, all of the studies on the effects of GCs in murine asthma models have evaluated the effect of GC treatment during the sensitization phase or the first challenge (see Section 5.1), not providing information about the effects of GCs during the established disease.

In the earlier mentioned study on the effects of GCs on Treg cell number in asthmatic horses [121], asthmatic horses and aged-match controls were exposed to allergens by being stabled and fed hay. After one month, they were treated with dexamethasone (0.06 mg/kg) once daily for two weeks, and the BALF was evaluated. The treatment increased the percentage of FoxP3^+^ T cells within CD4^+^ T cells significantly, from about 40% to about 65%; thus, the GCs did induce an increase in the Treg subset in the equine asthma model.

We also found a few studies on the effects of GCs in established asthma in humans. In one of these studies, Karagiannidis et al. demonstrated that topical GC treatment increased the FoxP3 mRNA level in the PBMCs of patients with moderate asthma by 2.2-fold compared to GC-untreated patients with moderate asthma [98]. Additionally, in the GC-treated patients, the levels of FoxP3 were correlated with the levels of IL-10 and TGF-β; thus, it seems that the cytokines were produced by the Treg subset or favored pTreg differentiation. In another study, the number of CD4^+^CD25^high^ T cells was found to be lower in the BALF of asthmatic children than in the BALF of children with a cough or control subjects, and in the children with asthma, inhaled corticosteroid treatment was associated with an increase in the percentage of CD4^+^CD25^high^ T cells in PBMCs and BALF. GCs were also found to restore the suppressive activity of Treg cells from asthmatic subjects [132]. 

In an OVA-induced murine model of asthma, in which the mid-term (six weeks after sensitization) effects of GC treatment were studied, the decrease in the Treg cell percentage (which was observed after GC treatment during the early phase, as described in Section 5.1) was no longer observed [123]. This confirms that in murine models, too, long-term treatment with GC may result in an increase in the Treg subset population.

In conclusion, the treatment of asthma patients with GCs not only produces anti-inflammatory effects, but also increases the number of Treg cells in the long term, possibly explaining why GCs are the most prescribed drugs in asthma and prevent the damage of lung parenchyma in the coming years. 

### 6.3. Effects on Multiple Sclerosis

The effects of GCs on multiple sclerosis patients in relapse are unclear. On the one hand, Braitch et al. demonstrated that there was a significant increase in the percentage of CD4^+^CD25^high^ Treg cells in PBMCs after the administration of intravenous methylprednisolone for two days, as well as a slight increase in the FoxP3/CD3 mRNA ratio [141]. On the other hand, Muls et al. demonstrated that the number of tTreg (evaluated as cells with the demethylation of the first intron of FoxP3) and of CD4^+^CD25^high^FoxP3^+^ cells had decreased following five days of treatment with intravenous methylprednisolone [142]. The differences in the results by the two studies are probably associated with differences in the total dose/schedule of methylprednisolone [142]. Moreover, the latter study demonstrated that the expression of CD39, an activation marker of Treg cells, was increased after GC treatment, indicating that GCs favor the maturation of the CD39^+^ pTreg cell subset.

In an interesting study on the effects of GCs in an experimental autoimmune encephalomyelitis mouse model of multiple sclerosis [131], the authors demonstrated that the beneficial effects of GCs in the model mice reconstituted with homozygous GR knockout fetal liver cells are attributable to the effects of GCs on peripheral T cells. They also demonstrated that the percentage of CD4^+^GITR^+^FoxP3^+^ Treg cells among CD4^+^ splenocytes is decreased by GC treatment, confirming the results reported by Muls et al.

It must be noted that experts are not in agreement about the role of Treg cells in immune-related diseases of the brain. For example, Baruch et al. demonstrated that in an Alzheimer disease model, the decrease in FoxP3^+^ Treg cells in the periphery is actually beneficial [150]. Thus, the findings that GC treatment reduces the number of Treg cells might represent a therapeutic effect of GCs.

### 6.4. Effects on Skin Diseases

One study has described the effect of topical GC treatment on the skin of psoriasis patients. In this study, 12 patients were treated with calcipotriol–betamethasone dipropionate ointment for eight weeks [140], and the number of FoxP3^+^ Treg cells was evaluated in biopsy samples taken before and after the treatment. The results demonstrate that with the exception of one patient, the number of FoxP3^+^ Treg cells was lower after the treatment, with a mean three-fold decrease, as was the ratio of FoxP3^+^/CD4^+^ cells, with a two-fold decrease. These findings demonstrate that local GC treatment decreases the number and percentage of Treg cells. Thus, the effects of GCs depend on the tissue and disease, and in psoriasis, the use of GCs may be deleterious in the long term. 

The above-described effects appear to be disease-specific more than tissue-specific. In fact, in Ni-allergic patients, treatment with systemic GCs was found to increase the number of dermal FoxP3^+^CD25^+^ Treg cells [113].

## 7. Effect of GCs on Treg Cell Number: In Vivo Findings in Tumors

Several years ago, it was proposed that GC treatment favors tumor growth and inhibits the cytotoxic effects of anti-tumoral drugs, and several findings have confirmed this conclusion. The mechanisms by which GCs exert their modulatory effects on tumor growth are several. In the beginning, attention was focused on the protective, anti-apoptotic effects of GC on non-lymphoid tumors [151,152]. Later on, several studies explained the effects of endogenous and exogenous GCs not only on the basis of their activity on tumor cells, but also of their immunosuppressive effects, both favoring tumor development [117,153,154] and hampering the response to anti-tumoral drugs [155,156,157]. Therefore, although GCs are still prescribed in patients with cancers for several reasons (i.e., to prevent emesis and hypersensitivity or allergic reactions to anti-tumor drugs, decrease fatigue, and stimulate appetite), it is becoming clear that the use of GCs may be dangerous when prescribed to patients with non-lymphoid tumors.

The studies on the immunosuppressive effects of GCs focus mainly on the effects on CD4^+^ and CD8^+^ T cells than on Treg cells. Moreover, existing results on Treg cell number are very few and contrasting. 

An elegant study evaluated the number of pulmonary metastasis following the injection of B16 cells through the lateral tail vein of mice in which sleep deprivation was induced, thus leading to increased concentrations of endogenous GCs [154]. Lung metastases appeared first in sleep-deprived mice than on control mice, and were in a higher number at the different time points. Of note, the number of CD8^+^ and NK cells was lower in the metastasis of sleep-deprived mice than control mice, and the conventional/Treg cell ratio was lower seven days after B16 cell injection, meaning that Tregs cells were increased relative to CD4^+^ conventional T cells in sleep-deprived mice. The study suggests that high levels of endogenous GCs decrease immunosurveillance and favor tumor development. In a well-established anti-PD-1-responsive murine tumor model, GC treatment was found to diminish the efficacy of anti-PD-1 therapy, with responses correlating with peripheral CD8^+^/Treg cell ratio [155]. The study suggests that, at least in this experimental model, treatment with GCs decreased the efficacy of treatment with immune checkpoint inhibitors and the effect was also due to the GC-dependent increase of Treg cells. 

The PBMCs of patients with malignant pleural mesothelioma were analyzed before and after receiving three 4-mg doses of dexamethasone (one dose every 12 h) in the 24 h prior to undergoing pemetrexed C platinum (carboplatin or cisplatin) chemotherapy [156]. The authors observed a large-scale lymphodepletive effect of dexamethasone, affecting CD4^+^ and CD8^+^ T cell subsets, so that the proportion of Treg cells within the CD4^+^ compartment was similar. Actually, the CD8^+^/Treg cell ratio was even increased. However, a significant increase in proliferation (percentage of Ki67^+^ Treg cells) and activation (percentage of ICOS^+^ Treg cells) of Treg cells was observed after dexamethasone was administered, so that the final effect of dexamethasone treatment on Treg cells was not clear. 

Mathuraja et al. studied the Treg cell (CD4^+^CD25^high^FoxP3^+^) number in multiple myeloma patients treated with lenalidomide and glucocorticoids [158]. The absolute number (median) and the percentage within CD4^+^ T cells (median) increased progressively after the treatment cycles, reaching a significant difference after three to four cycles, where there were about 50% more Treg cells than observed before treatment. The data are in agreement with the effects seen on mice following combined treatment with thalidomide (a drug structurally related to lenalidomide) and dexamethasone [159], and confirm the main role of GCs on Treg numbers. However, the Treg cell increase was higher in patients responding to the treatment, raising the issue of the role of Treg cells in multiple myeloma. Three years after, Scott et al. performed a study similar to the above and reached an opposite conclusion [160]. The mean percentage of Treg cells (CD4^+^CD25^high^FoxP3^+^) within CD4^+^ cells was much higher in the PBMCs of multiple myeloma patients than in healthy subjects, and decreased substantially after the first cycle with lenalidomide and glucocorticoids, even if the decrease was not significant.

In conclusion, studies on the effects of GCs on Treg cell number in patients with tumors are few and contrasting. Moreover, they evaluate the number of Treg cells in peripheral organs or PBMCs more than within the tumors, where changes would have a higher impact. Therefore, more studies are needed.

## 8. Key Signaling Molecules that Determine Treg Survival/Expansion/Differentiation

To understand the mechanism by which GCs promote the survival/expansion/differentiation of Treg cells, we must take into account (1) the specific phenotypic properties of these cells, (2) the differences in the signaling elicited by GCs on conventional T cells and on Treg cells, and (3) the signaling activated by GCs on non-T cells that promote Treg cell survival/expansion/differentiation through cytokine production.

In previous Sections, we have mentioned that in several contexts Treg cells have a weaker tendency to undergo GC-induced apoptosis than conventional T cells, which leads to an increase in the Treg/conventional T cell ratio. The reason for the selective protection of Treg cells is that, as demonstrated in mice, CD4^+^CD25^+^ T cells express the anti-apoptotic protein Bcl-2 at higher levels than CD4^+^CD25^−^ T cells [101]. Indeed, Huang et al. have shown that Bcl-2 can counteract GC-induced apoptosis in T lymphocytes [161].

It has been shown that TGF-β signaling is crucial for the induction of FoxP3 expression in naïve T cells and the generation of pTreg cells from conventional T cells [162,163]. There is also some evidence for the underlying mechanism: the activation of TGF-β receptors leads to the phosphorylation and nuclear translocation of SMAD proteins, and phosphorylated SMAD2 and SMAD3 bind to the FoxP3 promoter and work in synergy to induce FoxP3 expression during the conversion of naïve T cells [164]. One of the few genes induced by GCs in almost all cells is the glucocorticoid-induced leucine zipper (GILZ) gene [165]. Recently, Bereshchenko et al. demonstrated that GILZ was the link between GCs and the regulation of β-dependent pTreg differentiation from naïve T cells [166]. GILZ increased FoxP3 expression in naive CD4^+^CD25^−^ T cells, thus increasing the activation of the SMAD2 protein on stimulation with TGF-β [166].

GILZ has also been found to mediate the effects of GCs on major participants in the inflammatory and immune responses [167]. For example, GILZ plays a role in the inhibition of the NF-κB [168], AP-1 [169], and MAP kinase family pathways [170,171]. These signaling molecules also participate in T-cell differentiation, as reported by Barrat et al., who demonstrated that following combined treatment with dexamethasone and vitamin D3, NF-κB and AP-1 activities were inhibited in human and mouse naive CD4^+^ T cells that were in the process of differentiating into IL-10-producing Treg cells [91]. GCs also modulate the survival/expansion/differentiation of Treg cells indirectly through their effects on non-T cells. In 2007, Ito et al. identified a mechanism by which plasmacytoid dendritic cells (pDCs) generated IL-10-producing Treg cells through TLR-dependent and TLR-independent pathways [172]. Further, Grohmann et al. demonstrated that in vivo GC treatment results in an increase in the amount of GITR expressed by T cells and GITRL expressed by pDCs, and that GITRL activates non-canonical NF-κB signaling and IDO expression [173]. This was confirmed by other authors, who concluded that GC treatment conferred immunoregulatory properties on pDCs that were dependent on GITR expression by the host and required functional IDO [174].

Another mechanism by which GCs indirectly promote Treg expansion is the increased synthesis of TGF-β by non-T cells. Stary et al. demonstrated that GCs enhanced the production of TGF-β by Langerhans cells, and thereby induced the expansion of Treg cells [113]. Moreover, Hou et al. in 2016 demonstrated that dexamethasone-treated MDSCs upregulated the expression of IL-10 and TGF-β through increased expression of the transcription factor Ets1 [114].

**Figure 2:**
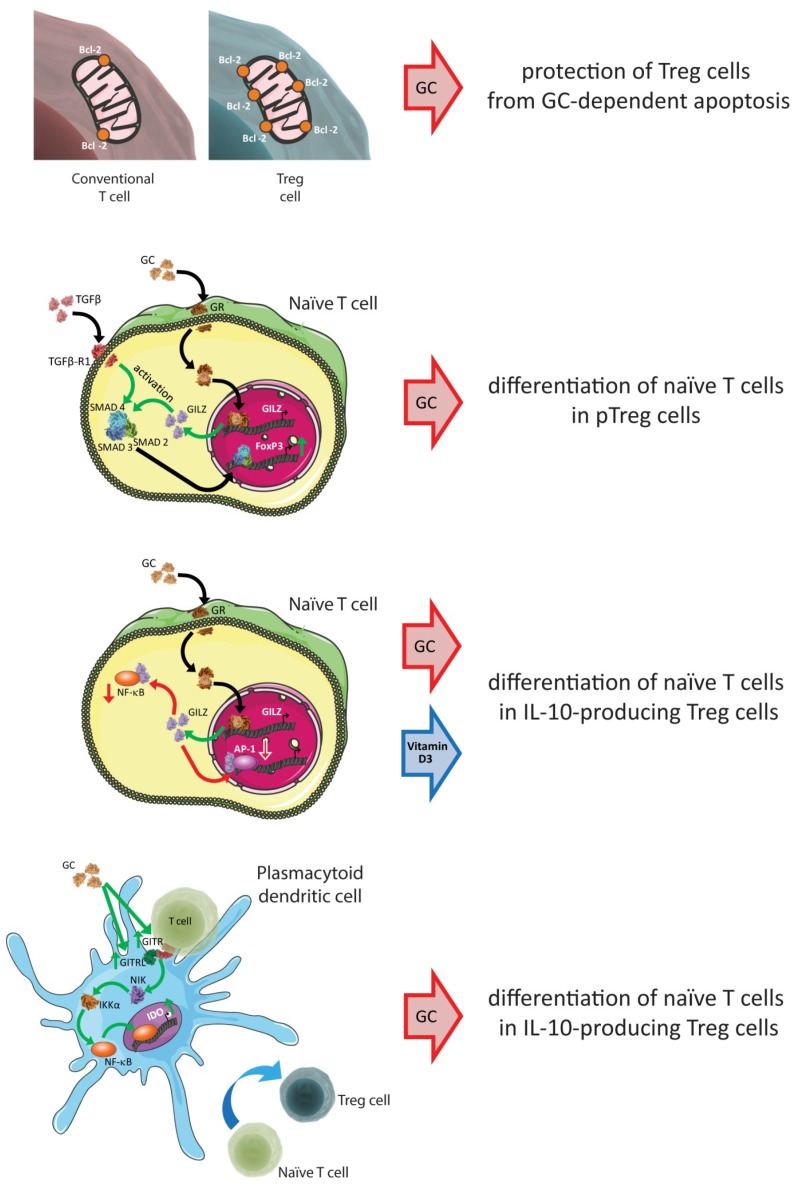
Key signaling molecules determining Treg cell survival/expansion/differentiation. The figure summarizes the key mechanisms by which GC treatment increases the number of Treg cells, which are described in detail in Section 8 of this review. Mechanisms include the increased expression by Treg cells of anti-apoptotic signals (upper panel), and the modulation of pathways in naïve T cells (middle panels) and non-T cells (lower panel). Red arrows indicate decreased expression and inhibition; green arrows indicate increased expression and activation.

In conclusion, GCs modulate the number of Treg cells, directly as well as indirectly, by modulating several pathways in T-cell subsets and in non-T cells (Figure 2). 

## 9. Concluding Remarks

According to several studies, the development and maintenance of autoimmune diseases is associated with a decrease in the Treg population or a defect in the suppressive activity of Treg cells. In other diseases, the partial resistance of conventional T cells to Treg cells has been described. In both instances, an increase in the number of fully active Treg cells may have beneficial effects in the long-term. For this reason, to know the effects of GC treatment on the absolute number of Treg cells and in relation with conventional T cells (percentage) appears crucial.

The in vitro data indicate that GCs favor the expansion of activated Treg cells, saving them from GC-induced apoptosis and favoring the differentiation of CD4^+^ T cells into pTreg cells. On the contrary, GCs determine the apoptosis of resting Treg cells, at least when evaluating the effect in experimental settings where Treg cells are cultured alone or with a few other cell types. Moreover, GCs promote the differentiation and expansion of pTreg cells indirectly, through the maturation/expansion of DCs, Langerhans cells, and MDSCs. 

In vivo studies confirmed that the induction of Treg cell expansion by GCs depends on the co-treatment conditions and whether or not the Treg cells are activated. GCs induced expansion in several conditions, including several autoimmune, allergic, and autoinflammatory diseases and in the response to grafts. However, GC treatment may exert pro-apoptotic effects on Treg cells when they are not activated (healthy or immunosuppressed subjects, for example), and may not change the Treg/conventional T cells ratio or even decrease the percentage of Treg cells. Interestingly, the inhibition of Treg cell expansion was demonstrated during allergen immunotherapy in asthmatic children and during the development of respiratory tolerance in a murine model, suggesting that GC should not be given during immunotherapy in patients who are allergic to respiratory allergens, and that the administration of systemic or topical GC to allergic children may hamper the maturation of the immune system, particularly the physiological tolerogenic response to respiratory allergens. On the contrary, the number of Treg cells was increased when GCs were given when the tolerogenic response happened in districts different from the respiratory system, such as skin and colon, or towards self and non-self antigens.

It appears that the effects of GCs on Treg numbers in patients with autoimmune and allergic diseases are disease-specific. Treg cell numbers increased in GC-treated patients with SLE, ITP, asthma, and allergy to nickel. On the contrary, the Treg cell numbers decreased in GC-treated patients with psoriasis. The effects of high doses of GCs are not clearly defined when used to treat multiple sclerosis patients in relapse.

In conclusion, the effects of GC treatment on the number of Treg cells in treated patients are different depending on the disease and possibly on the tissue. Considering that Treg cell expansion may play a relevant role in the disease control and development, the effect of GC on Treg numbers must be considered when planning treatment with GCs. Further studies are needed in order to study the effects of GC treatment in the autoimmune and autoinflammatory diseases where this effect was not investigated.

## Figures and Tables

**Table 1:** Modulation of regulatory T (Treg) cell subsets following GC treatment in healthy animals and disease models.

Paper	Species (Strain)	Disease	Drug, Dose, and Days of treatment		Time Elapsed from the Last Treatment	Evaluated Tissue	Treg Population	Modulation of the Treg Subset (Significance) ^1^
Boivin et al. 2018 [121]	Horse (N.A.)	No disease	14 day treatment with dexamethasone,.06 mg/Kg per day		on treatment	Bronchoalveolar lavage fluid	% FoxP3^+^ in CD4^+^ T cells	= vs. baseline
Chen et al. 2004 [101]	Mouse (BALB/c)	No disease	1 day treatment with dexamethasone, i.p., 5 mg/Kg		1 day after the injection	Thymus	CD4^+^CD25^+^ absolute number	↑(*) vs. untreated controls
							CD4^+^CD25^+^/CD4^+^CD25^−^ ratio	↑(**) vs. untreated controls
						Spleen	CD4^+^CD25^+^/CD4^+^CD25^−^ ratio	↑(**) vs. untreated controls
					3 days after the injection	Thymus	CD4^+^CD25^+^ absolute number	↑(*) vs. untreated controls
							CD4^+^CD25^+^/CD4^+^CD25^−^ ratio	↑(*) vs. untreated controls
			1, 3 and 5 day treatment with dexamethasone, i.p., 5 mg/Kg per day		1 day after the first injection	Thymus	CD4^+^CD25^+^ absolute number	↑(*) vs. untreated controls
							CD4^+^CD25^+^/CD4^+^CD25^−^ ratio	↑(*) vs. untreated controls
					1 day after the fifth injection	Thymus	CD4^+^CD25^+^ absolute number	↑(**) vs. untreated controls
							CD4^+^CD25^+^/CD4^+^CD25^−^ ratio	↑(**) vs. untreated controls
			3 day treatment with dexamethasone, i.p., 5 mg/Kg per day		1 day after the last injection	Thymus	CD4^+^CD8^−^CD25^+^ absolute number	↑(**) vs. untreated controls
						Spleen		↑(*) vs. untreated controls
						Lymph nodes		↑(*) vs. untreated controls
Chen et al. 2006 [120]	Mouse (BALB/c)	No disease	1–5 day treatment with dexamethasone, i.p., 5 mg/Kg per day plus IL-2 300 000 IU per day		1 day after the last injection	Spleen, lymph nodes	% CD25^+^ in CD4^+^ T cells	↑(**) vs. untreated controls
			3 day treatment with dexamethasone, i.p., 5 mg/Kg per day		1 day after the last injection	Spleen	CD4^+^CD25^+^/CD4^+^CD25^−^ ratio	↑(**) vs. untreated controls
							CD4^+^CD25^+^ absolute number	↓(*) vs. untreated controls
						Lymph nodes, spleen, blood	% CD4^+^FoxP3^+^ in all cells	↓(N.A.) vs. untreated controls
			3 day treatment with dexamethasone, i.p., 5 mg/Kg per day plus IL-2 300 000 IU per day		1 day after the last injection	Spleen	CD4^+^CD25^+^/CD4^+^CD25^−^ ratio	↑(**) vs. untreated controls
							CD4^+^CD25^+^ absolute number	↑(**) vs. untreated controls
							% CD4^+^FoxP3^+^ in all cells	↑(N.A.) vs. untreated controls
						Lymph nodes, blood	% CD4^+^FoxP3^+^ in all cells	↑(N.A.) vs. untreated controls
						Lymph nodes, spleen, blood	% FoxP3^+^ in CD3^+^CD4^+^ T cells	↑(N.A.) vs. untreated controls
					3 days after the last injection	Lymph nodes, spleen, blood	% FoxP3^+^ in CD3^+^CD4^+^ T cells	↓(N.A.) vs. untreated controls
					21 days after the last injection	Lymph nodes, spleen, blood	% FoxP3^+^ in CD3^+^CD4^+^ T cells	↑(N.A.) vs. untreated controls
Chen et al. 2018 [117]	Mouse (C57BL/6)	No disease	3 day treatment with dexamethasone, i.p. 0.1 or 100 μg per day		2 days after the last injection	Spleen	CD4^+^CD25^+^ absolute number	↑(*) or ↑ (***) (0.1, 100μg respectively) vs. untreated control
Sbiera et al.2011 [119]	Mouse (C57Bl/6)	No disease	3 day treatment with dexamethasone, i.p., 0.8 mg/Kg per day (similar results with 4, 20, 100 mg/Kg per day)		After treatment	Spleen, blood	CD4^+^CD25^high^FoxP3^+^ absolute number	↓(N.A.) vs. untreated controls
							% CD25^high^FoxP3^+^ in CD4^+^ T cells	↓(N.A.) vs. untreated controls
Zuska-Prot et al. 2017 [123]	Mouse (BALB/c)	No disease	9 day treatment with methylprednisolone (MP), i.m., 2 mg/Kg per day or 9 day treatment with Ciclesonide (CIC), inhaled, 160 μg per day		1 day after the last treatment	Lung	CD4^+^CD25^+^FoxP3^+^ absolute number	CIC↓(***) vs. untreated controls
						mediastinal lymph nodes		CIC↓(***) vs. untreated controls MP ↓(***) vs. untreated controls
						head and neck lymph nodes		CIC↓(***) vs. untreated controls MP ↓(***) vs. untreated controls
						blood		CIC↓(***) vs. untreated controls MP↓(*) vs. untreated controls
Ugor et al. 2018 [118]	Mouse (BALB/c)	No disease	1-4 day treatment with dexamethasone, i.p., 20 mg/Kg per day		1 day after the last injection	thymus	% CD25^+^FoxP3^+^ in CD4^+^ T cells	↑(N.A.) at day 1 vs. untreated controls (same result at day 2 and 4)
			1 day treatment with dexamethasone, i.p., 20 mg/Kg per day		4 or 8 h after the injection	blood	% CD25^+^FoxP3^+^ in CD4^+^ T cells	↑(*) vs. untreated controls
			4 day treatment with dexamethasone, i.p., 20 mg/Kg per day		1 day after the last injection	thymus	CD4^+^CD25^+^FoxP3^+^ absolute number	= vs. untreated controls
						spleen		↓(**) vs. untreated controls
						lymph nodes		↓(***) vs. untreated controls
						peyer’s patches		↓(*) vs. untreated controls
Kawalkowska et al. 2016 [124]	Mouse (DBA/1)	Arthritis	10 day treatment with dexamethasone, i.p., 160 μg per dayplus IL-4, i.p., on day 1, 4, and 7 post disease onset		on treatment	joints of paw	% CD25^+^FoxP3^+^ in CD4^+^ T cells	↑(**** vs. untreated controls) ↑(* vs. mice treated with Dex alone)
					on treatment	joints of paw	Th17/Tregs ratio	↓(* vs. untreated controls) ↓(** vs. mice treated with Dex alone)
					11 days after the last treatment			
Boivin et al. 2018 [121]	Horse (N.A.)	Severe asthma	14 day treatment with dexamethasone, 0.06 mg/Kg per day		on treatment	bronchoalveolar lavage fluid	% FoxP3^+^ in CD4^+^ T cells	↑(*** vs. healthy controls) ↑(* vs. baseline)
Olsen et al. 2015 [125]	Mouse (A/J)	Asthma (sensitization and first OVA challenges)	• (protocol 1) treatment with dexamethasone, os, 1 mg/Kg, same days of challenge (3 days/week during 3 weeks) • (protocol 2) treatment with dexamethasone, os, 1 mg/Kg per day, (on the last week of challenge) • (protocol 3) treatment with budesonide, nebulized, 7.5 mg/mL, same days as protocol 2, 3 (inhalation cycles of 10 min each) • (protocol 4) treatment with budesonide, nebulized, 7.5 mg/mL, same days as protocol 2, 3 (inhalation cycles of 30 min each)	protocol 1, protocol 2, and protocol 3	1 day after the last treatment	bronchoalveolar lavage fluid	CD4^+^CD25^+^FoxP3^+^ absolute number	↓(*) vs. untreated mice
						lung		
				protocol 1	7 days after the last treatment	bronchoalveolar lavage fluid		
				protocol 1, protocol 2, and protocol 3	1 day after the last treatment	lymph node		
				protocol 2, and protocol 3	1 day after the last treatment	thymus		
				protocol 2 and protocol 4	1 day after the last treatment	airways and lung	CD4^+^CD25^+^FoxP3^+^ absolute number	↓(*) vs. untreated mice
	Mouse (BALB/c)	Asthma (HDM challenges)		protocol 2 and protocol 4	1 day after the last treatment	airways and lung	CD4^+^CD25^+^FoxP3^+^ absolute number	↓(*) vs. untreated mice
Wu et al. 2016 [126]	Mouse (BALB/c)	Asthma (sensitization and first OVA challenges)	3 day treatment with dexamethasone, ranging from 12.5 to 18.75 μg/day plus IL-2, intratracheal, ranging from 50000 to 75000 IU per day		1 day after the last treatment	bronchoalveolar lavage fluid	CD4^+^CD25^+^ absolute number	↑(*) vs. untreated asthmatic mice
Zou et al. 2017 [127]	Mouse (BALB/c)	Asthma (sensitization and first OVA challenges)	7 day treatment with dexamethasone, i.p., 1 mg/kg per day		N.A.	bronchoalveolar lavage fluid	% CD25^+^FoxP3^+^ in CD4^+^ T cells	↓(**) vs. healthy controls ↑(N.A.) vs. asthmatic untreated mice
						pulmonary tissue	FoxP3 expression (evaluated by q-PCR, IHC, and SDS-PAGE)	↓(**) vs. healthy controls ↑(N.A.) vs. asthmatic untreated mice
Zuska-Prot et al. 2017 [123]	Mouse (BALB/c)	asthma (sensitization and first OVA challenges)	treatment with Ciclesonide (CIC), inhaled, 160 μg/mouse per day or treatment with methylprednisolone (MP), i.m., 2 mg/kg per day	4 days of treatment	on treatment	lung	CD4^+^CD25^+^FoxP3^+^ absolute number	OVA+CIC↓(***) vs. untreated controls OVA+MP↓(**) vs. untreated controls
						Mediastinal lymph nodes		OVA+CIC↓(*) vs. untreated controls and healthy controls
				9 days of treatment	on treatment	lung	CD4^+^CD25^+^FoxP3^+^ absolute number	OVA+CIC↓(***) vs. untreated controls OVA+MP↓(**) vs. untreated controls
						mediastinal lymph nodes		OVA+CIC↓(***) vs. untreated controls OVA+MP↓(*) vs. untreated controls
						head and neck lymph nodes		OVA+CIC↓(***) vs. untreated controls and healthy controls OVA+MP↓(***) vs. untreated controls and healthy controls
						peripheral blood		OVA+CIC↓(***) vs. healthy controls OVA+MP↓(***) vs. healthy controls
				23 days of treatment	on treatment	lung	CD4^+^CD25^+^FoxP3^+^ absolute number	OVA+CIC↓(***) vs. untreated controls OVA+MP↓(***) vs. untreated controls
						mediastinal lymph nodes		OVA+CIC↓(***) vs. healthy controls and untreated controls OVA+MP↓(***) vs. healthy controls and untreated controls
						head and neck lymph nodes		OVA+CIC↓(***) vs. untreated controls and healthy controls OVA+MP↓(***) vs. healthy controls
						peripheral blood		OVA+CIC↓(***) vs. healthy controls and untreated controls OVA+MP↓(***) vs. healthy controls and untreated controls
Boivin et al. 2018 [121]	Horse (N.A.)	severe asthma	14 day treatment with dexamethasone, 0.06 mg/Kg per day		on treatment	bronchoalveolar lavage fluid	% FoxP3^+^ in CD4^+^ T cells	↑(*** vs. healthy controls) ↑(* vs. baseline)
Chen et al. 2014 [128]	Mouse (APOE^−/−^ C57BL/6)	atherosclerosis	3 day treatment with dexamethasone, i.m., 4.5 mg/Kg on day 1, 2.25 mg/Kg on day 2 and 3		14 days after the last treatment	spleen	% FoxP3^+^ in CD4^+^ T cells	↑(*) vs. untreated controls
Nakao et al. 2018 [116]	Mouse (B6N)	cardiac graft	6 day treatment with dexamethasone, i.p., 5 mg/Kg on day 0,2,4, and 6)		4 days after the last treatment	spleen	% FoxP3^+^ in CD3^+^CD4^+^	↑(*) vs. untreated controls
							% FoxP3^+^ in splenocytes	↑(***) vs. untreated controls
			6 day treatment with dexamethasone, i.p., 5 mg/Kg on day 0,2,4, and 6), plus anti-Gr-1 Ab, i.p., on postoperative days 1 and 4				% FoxP3^+^ in CD3^+^CD4^+^	↓(***) vs. mice treated with dexamethasone alone
							% FoxP3^+^ in splenocytes	↓(***) vs. mice treated with dexamethasone alone
You et al. 2018 [129]	Mouse (BALB/c)	colitis	4 day treatment with dexamethasone, orogastric gavage, 5 mg/Kg per day, plus AdTGF^2^		N.A.	mesenteric lymph nodes	FoxP3 expression (q-PCR)	↑(**) vs. mice treated with AdTGF-1 alone
							FoxP3^+^ absolute number	
Zhang et al. 2013 [130]	Mouse (NOD)	type I diabetes	(14-days protocol) treatment with dexamethasone in the two hind footpads, 16 mg/Kg on days 1, 4, 7, 10, plus injection of insuline peptide (B9-23) co-injected at day-7		7 days after the last treatment	spleen	% CD25^+^FoxP3^+^ in CD4^+^ T cells	↑(*) vs. untreated controls, mice treated with dexamethasone alone and insuline peptide alone
							% FoxP3^+^IL-10^+^ in CD4^+^CD25^+^ T cells	
					45 days after the last treatment		% CD44^+^CD62L^−^ in CD4^+^FoxP3^+^ T cells	↑(*) vs. mice treated with insulin peptide
Chen et al. 2006 [120]	Mouse (C57BL/6)	experimental autoimmune encephalomyelitis (EAE)	3 day prior to immunization treatment with dexamethasone, i.p., 5 mg/Kg plus IL-2, i.v., 4 μg		N.A.	spleen	% FoxP3^+^ in CD4^+^ T Cells	↑(N.A.) vs. untreated EAE mice
Wüst et al. 2008 [131]	mouse ^3^ (C57BL/6 Gr^flox^)	experimental autoimmune encephalomyelitis (EAE)	3 day treatment with dexamethasone, i.p., 100 mg/Kg per day		58 h after the last treatment	spleen	% FoxP3^+^GITR^+^ in CD4^+^ Tcells	↓(N.A.) vs. untreated controls
							MFI of FoxP3	↓(**) vs. untreated controls
	mouse ^3^ (C57BL/6 Gr^lck^)						% FoxP3^+^GITR^+^ in CD4^+^ T cells	↓(N.A.) vs. untreated controls
							MFI of FoxP3	= vs. untreated controls

^1^ ↑, increase; (*) *p* < 0.05, (**) *p* < 0.01, (***) *p* < 0.001, (****) *p* < 0.0001, (N.A.), not available; ↓, decrease; (*) *p* < 0.05, (**) *p* < 0.01, (***) *p* < 0.001, ( N.A.) not available; ^2^ adenovirus expressing TGF-β; ^3^ GR^lck^ mice, the T cells of these mice do not express the glucocorticoid receptor; Gr^flox^, control mice.

**Table 2:** Modulation of human Treg cell subsets following GC treatment in health and diseases.

Paper	Disease	Drug, Dose, and Days of Treatment	Time Elapsed from the Last Treatment	Evaluated Tissue	Treg Population	Modulation of the Treg Subset (Significance) ^1^
Sbiera et al. 2011 [119]	No disease	(14 days protocol) treatment with prednisolone, i.v., 250 μg/day, on days 1–3 i.v., 150 μg on day 4 os, 100 μg/day on days 5–9 os, 75 μg/day on days 10–11 os, 50 μg on day 12 os, 20 μg on day 13 os, 10 μg on day 14	on treatment	peripheral blood mononuclear cells	% FoxP3^+^ in CD4^+^ T cells	↓(*) vs. baseline
					CD4^+^CD25^high^FoxP3^+^ absolute number	↑(*) vs. baseline
					% CD127^−^ in CD4^+^ T cells	↑(*) vs. baseline
					CD4^+^CD25^high^FoxP3^+^CD127^−^ absolute number	↑(***) vs. baseline
					CD4^+^CD25^high^FoxP3^+^CTLA^+^ absolute number	↑(*) vs. baseline
Hartl et al. 2007 [132]	Asthma	28 day treatment with inhaled fluticasone, 0.4 μg/day	after treatment	blood, bronchoalveolar lavage fluid	% CD25^high^ in CD4^+^ T cells	↑(* blood and ** bronchoalveolar lavage fluid) vs. baseline
		at least 90 day treatment with inhaled fluticasone, 0.4 μg/day	on treatment	blood, bronchoalveolar lavage fluid	% CD25^high^ in CD4^+^ T cells	↑(* blood and ** bronchoalveolar lavage fluid) vs. untreated asthma patients
Karagiannidis et al. 2004 [98]	Severe asthma	treatment with inhaled fluticasone, 2 μg/day plus prednisolone, os, 42.2 μg/day	12h after the last treatment	blood	CD4^+^CD25^+^CD45RO^+^CD62L^+^ absolute number	↑(*) vs. baseline
			3h after the last treatment	peripheral blood mononuclear cells	FoxP3 expression in CD4^+^ T cells (q-PCR)	
			on treatment			↑(*) vs. healthy controls ↑(**) vs. untreated moderate asthma patients
	Moderate asthma	inhaled steroids	on treatment	peripheral blood mononuclear cells	% CD25^high^ in CD4^+^ T cells	↑(*) vs. healthy controls ↑(**) vs. baseline
Seissler et al. 2012 [133]	Kidney transplant	3 day treatment with methylprednisolone, 125 or 250 μg/day	on treatment	peripheral blood mononuclear cells	% DR^high^CD45RA^−^ in FoxP3^+^CD127^low/−^ Treg cells	↑(**) at day 1 and 2, vs. baseline ↑(*) at day 3 vs. baseline
					% DR^+^CD45RA^−^ in FoxP3^+^CD127^low/−^ Treg cells	↑(**) at day 1 vs. baseline ↑(*) at day 2 and 3 vs. baseline
					% DR^low^CD45RA^−^ in FoxP3^+^CD127^low/−^ Treg cells	↑(*) at day 1 vs. baseline
					% DR^−^CD45RA^+^ in FoxP3^+^CD127^low/−^ Treg cells	↓(**) at day 3 vs. baseline
Azab et al. 2008 [134]	Systemic lupus erythematosus (SLE)	at least 90 day treatment with glucocorticoids therapy (various doses)	on treatment	peripheral blood mononuclear cells	MFI of CD25 in CD4^+^CD25^+^ T cells	↑(*) vs. healthy controls
					MFI of CD25 in CD4^+^CD25^high^ T cells	↑(*) vs. healthy controls
					% CD25^+^ in CD4^+^ T cells	↑(*) vs. healthy controls and untreated patients
Mathian et al. 2015 [135]	Systemic lupus erythematosus (SLE)	3 day treatment with methylprednisolone, i.v., 500 or 1000 μg/day	on treatment	peripheral blood mononuclear cells	% FoxP3^bright^CD45RA^−^ in CD4^+^ T cells	↑(**) at day 1 and 3 vs. baseline ↑(***) at day 2 vs. baseline
Suarez et al. 2006 [136]	Systemic lupus erythematosus (SLE)	at least 90 day treatment with glucocorticoids therapy (various doses)	on treatment	blood	% CD25^high^ in CD4^+^ T cells	↑(*) vs. baseline ↑ (**) vs. healthy controls
Zhang et al. 2008 [137]	Systemic lupus erythematosus (SLE)	treatment with prednisolone, ≥1 μg/day plus intravenous cyclophosphamide, every 2–4 weeks	on treatment	peripheral blood mononuclear cells	% CD25^high^ in CD4^+^ T cells	↑(*) vs. baseline
Li et al. 2011 [138]	Immune thrombocytopenia	(28 days protocol) treatment with dexamethasone, os, 40 μg/day, on day 1–4 prednisone, os, 60 μg/day, on day 5–7 prednisone, os, 30 μg/day, on day 8–14 prednisone, os, 15 μg/day, on day 15–21 prednisone, os, 10 μg/day, on day 22–28	on treatment	blood	CD4^+^CD25^+^CD127^−^ absolute number	↑(**) vs. baseline ↓(**) vs. day 14 of treatment
Li et al. 2013 [99]	Immune thrombocytopenia	4 day treatment with dexamethasone, 40 μg/day	N.A.	peripheral blood mononuclear cells	FoxP3 expression (q-PCR)	↑(**) vs. untreated patients
					CD4^+^CD25^high^FoxP3^+^ absolute number	↑(*) vs. untreated patients
Ling et al. 2007 [139]	Immune thrombocytopenia	4 day treatment with dexamethasone, os, 40 μg/day	1 day after the last treatment	peripheral blood mononuclear cells	% FoxP3^+^ in CD4^+^ T cells	↑(****) vs. baseline
Keijsers et al. 2015 [140]	Psoriasis	56 day treatment with calcipotriol–betamethasone dipropionate ointment	after treatment	skin biopsies	FoxP3^+^ cells/mm^2^ (IHC)	↓(**) vs. baseline
					FoxP3/CD4 ratio (IHC)	↓(*) vs. baseline
Braitch et al. 2009 [141]	Multiple sclerosis (relapse)	3 day treatment with methylprednisolone, i.v., 1000 μg/day	on treatment	peripheral blood mononuclear cells	CD4^+^ CD25^high^ absolute number	↑(**) vs. baseline
					FoxP3/CD3 ratio (q-PCR)	↑(**) vs. baseline
Muls et al. 2015 [142]	Multiple sclerosis (relapse)	5 day treatment with methylprednisolone, i.v., 1000 μg/day	on treatment	peripheral blood mononuclear cells	% CD25^high^FOXP3^+^ in lymphoid cells	↓(****) vs. baseline
					% CD25^high^FOXP3^+^ in CD4^+^ T cells	↓(*) vs. baseline
					FoxP3 expression (q-PCR) in CD4^+^ T cells	↓(**) vs. baseline
					CD4^+^CD25^high^FOXP3^+^CD39^+^ absolute number	↑(*) vs. baseline

^1^ ↑, increase; (*) *p* < 0.05, (**) *p* < 0.01, (***) *p* < 0.001, (****) *p* < 0.0001; ↓, decrease; (*) *p* < 0.05, (**) *p* < 0.01, (****) *p* < 0.0001.

## Abbreviations

AP-1, Activator Protein 1; BALF, bronchoalveolar lavage fluid; Bcl-2, B-Cell Lymphoma 2; CTLA-4, Cytotoxic T-Lymphocyte Associated Protein 4; DCs, Dendritic Cells; DNBS, dinitrobenzene sulfonic acid; Ets1, ETS Proto-Oncogene 1 Transcription Factor; FoxP3, Forkhead Box P3; GCs, Glucocorticoids; GILZ, Glucocorticoid-Induced Leucine Zipper gene; GITR, Glucocorticoid-Induced Tumor Necrosis Factor Receptor-Related Protein; GITRL, Glucocorticoid-Induced Tumor Necrosis Factor Receptor-Related Protein Ligand; GITRsp, GITR single-positive cells; GR, Glucocorticoid Receptor; HLA-DR, Human Leukocyte Antigen-DR isotype; HSP60, Heat Shock Protein 60; ICOS, Inducible T-Cell Costimulator; IDO, Indoleamine 2,3-Dioxygenase 1; Itk, Interleukin 2-inducible T Cell Kinase; ITP, immune thrombocytopenic purpura; LAG-3, Lymphocyte-Activation Gene 3; MDSCs, myeloid-derived suppressor cells; NF-κB, Nuclear Factor of Kappa Light Polypeptide Gene Enhancer In B-Cells; NOD, non-obese diabetic mice; nTreg, natural Treg cells; OVA, ovalbumin; PBMCs, peripheral blood mononuclear cells; pDCs, plasmacytoid Dendritic Cells; pTreg, peripherally derived Treg; SLE, systemic lupus erythematosus; SMAD, small mother against decapentaplegic; T-bet, T-Box Expressed in T Cells; TGF-β, Transforming Growth Factor Beta; Th1, T helper type 1 cells; Th17, T helper type 17 cells; Th2, T helper type 2 cells; Th3, T helper type 3 cells; TLR, Toll-Like Receptor; Tr1, T regulatory type 1 cells; Treg, regulatory T cells; tTreg, thymus derived Treg.
